# RNA-Binding Proteins HuB, HuC, and HuD are Distinctly Regulated in Dorsal Root Ganglia Neurons from STZ-Sensitive Compared to STZ-Resistant Diabetic Mice

**DOI:** 10.3390/ijms20081965

**Published:** 2019-04-22

**Authors:** Cosmin Cătălin Mustăciosu, Adela Banciu, Călin Mircea Rusu, Daniel Dumitru Banciu, Diana Savu, Mihai Radu, Beatrice Mihaela Radu

**Affiliations:** 1 Department of Life and Environmental Physics, ’Horia Hulubei’ National Institute of Physics and Nuclear Engineering, Reactorului 30, 077125 Bucharest-Magurele, Romania; cosmin@nipne.ro (C.C.M.); calin.rusu@nipne.ro (C.M.R.); dsavu@nipne.ro (D.S.); 2 Department of Bioengineering and Biotechnology, Faculty of Medical Engineering, University Politehnica of Bucharest, Gheorghe Polizu Street 1-7, 011061 Bucharest, Romania; adela.banciu79@gmail.com (A.B.); danieldumitrubanciu@gmail.com (D.D.B.); 3 Department of Anatomy, Animal Physiology, and Biophysics, Faculty of Biology, University of Bucharest, Splaiul Independenţei 91-95, 050095 Bucharest, Romania; beatrice.radu@bio.unibuc.ro; 4 Life, Environmental and Earth Sciences Division, Research Institute of the University of Bucharest (ICUB), Splaiul Independenţei 91-95, 050095 Bucharest, Romania

**Keywords:** *Elav*-like, Hu proteins, diabetes, streptozotocin, thermal response, hypoalgesia, dorsal root ganglia neurons

## Abstract

The neuron-specific *Elav*-like Hu RNA-binding proteins were described to play an important role in neuronal differentiation and plasticity by ensuring the post-transcriptional control of RNAs encoding for various proteins. Although *Elav*-like Hu proteins alterations were reported in diabetes or neuropathy, little is known about the regulation of neuron-specific *Elav*-like Hu RNA-binding proteins in sensory neurons of dorsal root ganglia (DRG) due to the diabetic condition. The goal of our study was to analyze the gene and protein expression of HuB, HuC, and HuD in DRG sensory neurons in diabetes. The diabetic condition was induced in CD-1 adult male mice with single-intraperitoneal injection of streptozotocin (STZ, 150 mg/kg), and 8-weeks (advanced diabetes) after induction was quantified the *Elav*-like proteins expression. Based on the glycemia values, we identified two types of responses to STZ, and mice were classified in STZ-resistant (diabetic resistant, glycemia < 260 mg/dL) and STZ-sensitive (diabetic, glycemia > 260 mg/dL). Body weight measurements indicated that 8-weeks after STZ-induction of diabetes, control mice have a higher increase in body weight compared to the diabetic and diabetic resistant mice. Moreover, after 8-weeks, diabetic mice (19.52 ± 3.52 s) have longer paw withdrawal latencies in the hot-plate test than diabetic resistant (11.36 ± 1.92 s) and control (11.03 ± 1.97 s) mice, that correlates with the installation of warm hypoalgesia due to the diabetic condition. Further on, we evidenced the decrease of *Elav*-like gene expression in DRG neurons of diabetic mice (*Elavl2*, 0.68 ± 0.05 fold; *Elavl3*, 0.65 ± 0.01 fold; *Elavl4*, 0.53 ± 0.07 fold) and diabetic resistant mice *(Ealvl2*, 0.56 ± 0.07 fold; *Elavl3*, 0.32 ± 0.09 fold) compared to control mice. Interestingly, *Elav*-like genes have a more accentuated downregulation in diabetic resistant than in diabetic mice, although hypoalgesia was evidenced only in diabetic mice. The *Elav*-like gene expression changes do not always correlate with the Hu protein expression changes. To detail, HuB is upregulated and HuD is downregulated in diabetic mice, while HuB, HuC, and HuD are downregulated in diabetic resistant mice compared to control mice. To resume, we demonstrated HuD downregulation and HuB upregulation in DRG sensory neurons induced by diabetes, which might be correlated with altered post-transcriptional control of RNAs involved in the regulation of thermal hypoalgesia condition caused by the advanced diabetic neuropathy.

## 1. Introduction

Hu proteins are members of the RNA-binding proteins (RBP) superfamily and are encoded by Embryonic Lethal, Abnormal Vision, and Drosophila (*ELAV*) genes. The Hu proteins family has four members HuB (encoded by *ELAV-like 2* gene), HuC (encoded by *ELAV-like 3* gene), HuD (encoded by *ELAV-like 4* gene), and HuR or HuA (encoded by *ELAV-like 1* gene). Three of these proteins have identified as neuronal specific (i.e., HuB, HuC, and HuD), while the fourth is ubiquitary (HuR). 

RBP are well known for the post-transcriptional control of RNAs encoding multiple proteins [1]. In particular, RBPs play essential roles in the nervous system, such as alternative splicing of neuronal proteins (i.e., neurotransmitters, membrane receptors, cell adhesion molecules, and components of signal transduction proteins), protection of the mRNAs for long-distance transport and guidance of the protein localization [2,3,4,5]. 

Neuronal-enriched *ELAV*-like (*nELAVL*) Hu proteins were described to play essential roles in neuronal development and plasticity [6,7] in the central and peripheral nervous system. *nELAVL* Hu proteins are binding to the adenylate-uridylate-rich (ARE) RNA elements in the 3′ untranslated regions (3′-UTR) of target proteins, including growth associated protein 43 (GAP-43) [8,9], c-myc and vascular endothelial growth factor (VEGF) [10], and neprilysin (a potent amyloid β degrading enzyme) [11] stabilizing them. Moreover, *nELAVL* Hu proteins autoregulate themselves [12] or interact/stabilize other neuronal RBPs, e.g., Musashi-1 [13] and NOVA1 [14]. In the central nervous system, *nELAVL* Hu-proteins have been involved in regulating neuronal excitability by controlling the glutamate synthesis pathway and their gene deletion induces spontaneous epileptic seizure activity [15], by binding to the mRNA encoding Kv1.1 voltage-gated potassium channels [16]. In the peripheral nervous system, *nELAVL* Hu proteins are localized in the dorsal root ganglia (DRG) neurons [17,18,19,20]. This anatomical localisation of *nELAVL* Hu-proteins is correlated with their functional role of binding mRNA encoding proteins (i.e., brain-derived neurotrophic factor, GAP-43) involved in peripheral nerve regeneration upon lesion [8,21,22], being upregulated in the early stages of nerve recovery. 

The role of RBPs in diabetes and its complications was extensively documented [23]. To detail, it was described the regulation of beta-pancreatic cell function by various RBPs [24], including neuronal-enriched RBPs [25]. The altered regulatory function exerted by the ubiquitary HuR protein in diabetes was often described [25,26,27,28]. On the other hand, although the role of *nELAVL* Hu proteins was described in diabetes [29,30,31], yet no attention was paid so far to their expression changes in DRG sensory neurons associated with the diabetic condition. 

We aimed to elucidate the role played by Hu proteins expressed by the DRG sensory neurons in diabetes. To this purpose, we have employed the streptozotocin (STZ)-induced model of diabetes in CD-1 adult male mice. We have explored the gene and protein expression for *nELAVL* Hu proteins in DRG neurons between diabetic and control mice and we correlated them with the changes in animal glycemia, body weight, or their response to hot thermal stimulation. We also analyzed the distinct changes between animals sensitive or resistant to the STZ-induction of diabetes.

## 2. Results

### 2.1. Diabetic Mice Have Changes in Glycemia and Body Weight Compared to Diabetic Resistant or Control Mice 

**Figure 1:**
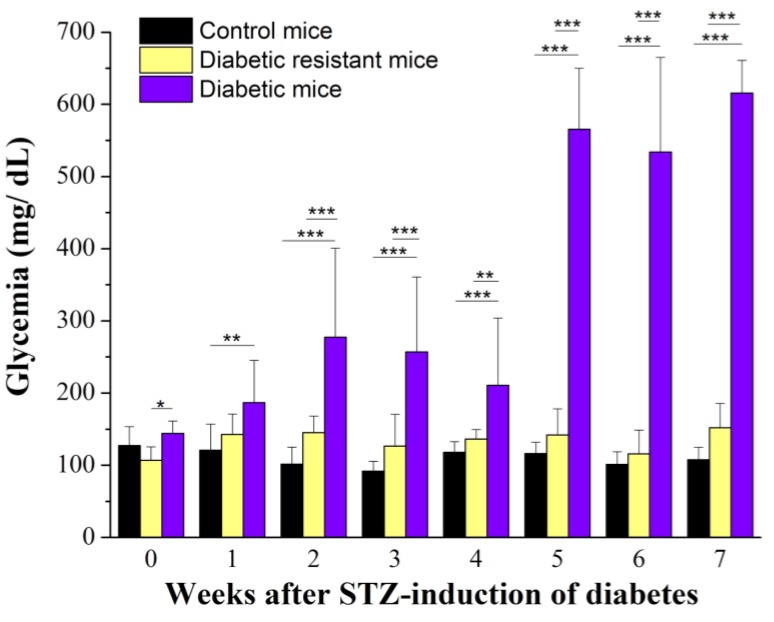
Blood glucose values (in mg/dL) were represented as mean ± SD for control, diabetic resistant, and diabetic mice. Statistical significance was indicated *** *p* < 0.001, ** *p* < 0.01, * *p* < 0.05.

We started our experimental protocol with two CD-1 mice groups: citrate buffer-injected group (*N* = 20) and STZ-injected group (*N* = 20). In the STZ-injected group, seven out of 20 animals died quickly. We measured the glycemia weekly for 7 weeks. Hyperglycemia was considered above 260 mg/dL, as previously described [32,33]. Considering the hyperglycemia threshold, at the end of 7 weeks we separated the surviving animals of the STZ-injected group (*N* = 13) into two subgroups: STZ-sensitive group (*N* = 7, glycemia > 260 mg/dL) and STZ-resistant group (*N* = 6, glycemia < 260 mg/dL), that will be further called diabetic group and diabetic resistant group, respectively. Then, we plotted the glycemia variation for the diabetic, diabetic resistant, and control mice groups (Figure 1). 

Only the diabetic group had an increase in glycemia (from 144.16 ± 17.29 mg/dL to 615.50 ± 45.05 mg/dL, *N* = 7), while the diabetic resistant group (from 106.85 ± 18.73 mg/dL to 152.14 ± 33.55 mg/dL, *N* = 6) and the control group (from 127.89 ± 26.63 mg/dL to 107.89 ± 36.63 mg/dL, *N* = 20) had no significant changes. The two-way ANOVA analysis indicated statistical significance of the glycemia, for the diabetic condition, and for their interaction (Table S1). The one-way ANOVA weekly comparison between the animal groups indicated that diabetic mice had higher glycemia compared to diabetic resistant and control mice, starting from the first week after STZ-induction of diabetes and with this difference accentuating to fifth–seventh week (Table S2) and is indicated in Figure 1. The weekly comparison in the diabetic group showed the increase of glycemia up to the fifh week, followed by a plateau-like evolution up to the eighth week (Table S3). Meanwhile, the weekly comparison of the glycemia values for the control and diabetic groups did not indicate significant changes. 

**Figure 2:**
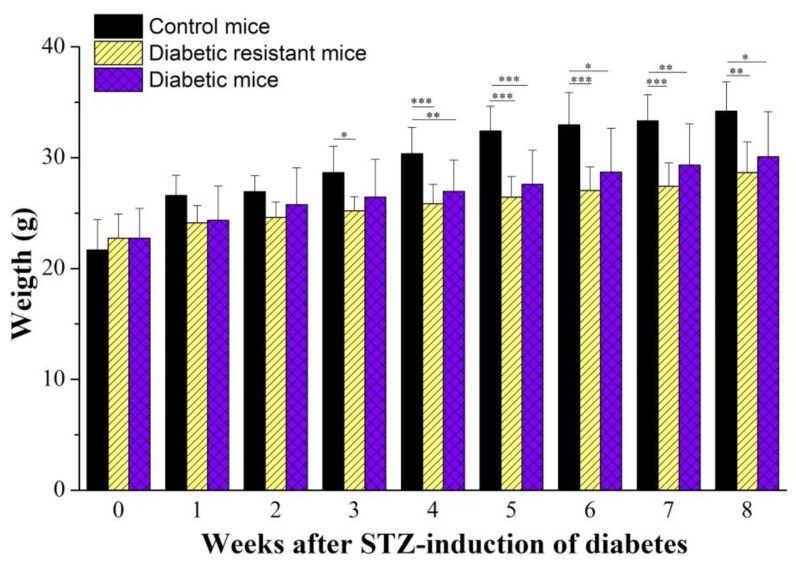
Body weight (in g) monitored for 8 weeks after streptozotocin (STZ)-induction of diabetes. Body weight values were represented as mean ± SD for control, diabetic resistant, and diabetic mice. Statistical significance is indicated * *p* < 0.05, ** *p* < 0.01, *** *p* < 0.001.

We have also measured the body weight for the diabetic, diabetic resistant and control CD-1 mice groups weekly, for 8 weeks, after the STZ-diabetes induction (Figure 2). All animal groups had an overall increase of the body weight, but the increase rate was higher for the control group (from 21.75 ± 2.75 g to 34.19 ± 2.66 g, *N* = 20) compared to the diabetic group (from 22.74 ± 2.68 g to 30.08 ± 2.66 g, *N* = 7) and diabetic-resistant group (from 22.74 ± 2.18 g to 28.65 ± 2.78 g, *N* = 6). The two-way ANOVA analysis indicated statistical significance of the body weight, of the diabetic condition, and of their interaction (Table S4). The one-way ANOVA comparison between the animal groups indicated that control mice were heavier than diabetic mice and diabetic resistant mice, starting from the fourth wk after STZ-induction of diabetes (Table S5). The one-way ANOVA weekly comparison in each animal group showed a continuous body weight increase, for the whole duration of the protocol (for 8 weeks), with statistical significance in all three animal groups (Table S6).

### 2.2. Diabetic Mice Have Longer Paw Withdrawal Latencies to Nociceptive Thermal Stimulation than Diabetic Resistant or Control Mice

**Figure 3:**
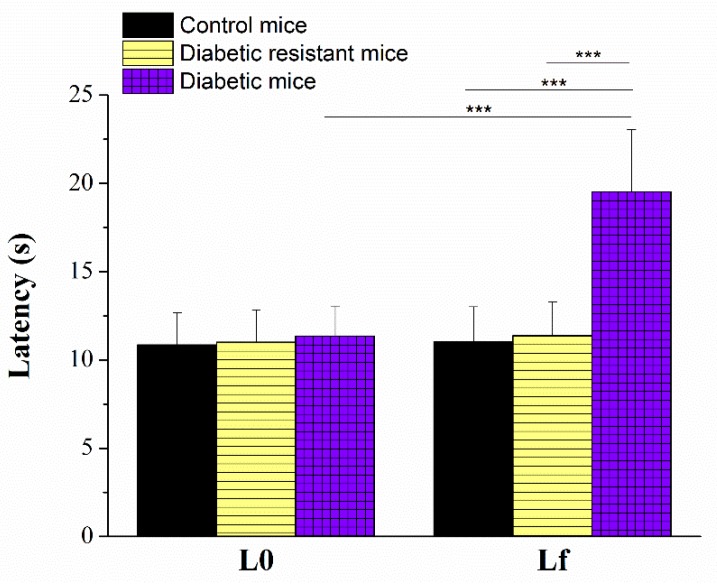
Paw withdrawal latencies (in s) in response to radiant heat (55 °C). Initial (L0) and final (Lf) withdrawal latencies were represented as mean ± SD for control, diabetic resistant, and diabetic mice. Statistical significance is indicated *** *p* < 0.001.

We employed the hot-plate test at the fixed temperature of 55 °C and we measured the paw withdrawal latency in order to evaluate the thermal response in diabetic, diabetic resistant, and control mice groups (Figure 3). The two-way ANOVA analysis indicated statistical significance of the latency, of the diabetic condition, and of their interaction (Table S7). We compared the initial paw withdrawal latency (L0) measured at the beginning of the protocol (before any treatment), with the final paw withdrawal latency (Lf) measured after 8 weeks of the STZ-induction of diabetes. In the diabetic group, the final latency (Lf = 19.52 ± 3.52 s, *N* = 7) is significantly longer than the initial latency (L0 = 11.35 ± 1.69 s, unpaired *t*-test, *p* < 0.001). On the other hand an intergroup comparison of the final latency showed a statistical significant increase in the diabetic group (19.52 ± 3.52 s, *N* = 7) compared to the diabetic resistant group (11.36 ± 1.92 s, *N* = 6) or to the control group (11.03 ± 1.97 s, *N* = 20) (Table S8). 

### 2.3. Elav-Like Gene Expression in Mouse DRG Neurons Is Decreased in Diabetic and Diabetic Resistant Mice Compared to Control Mice

**Figure 4:**
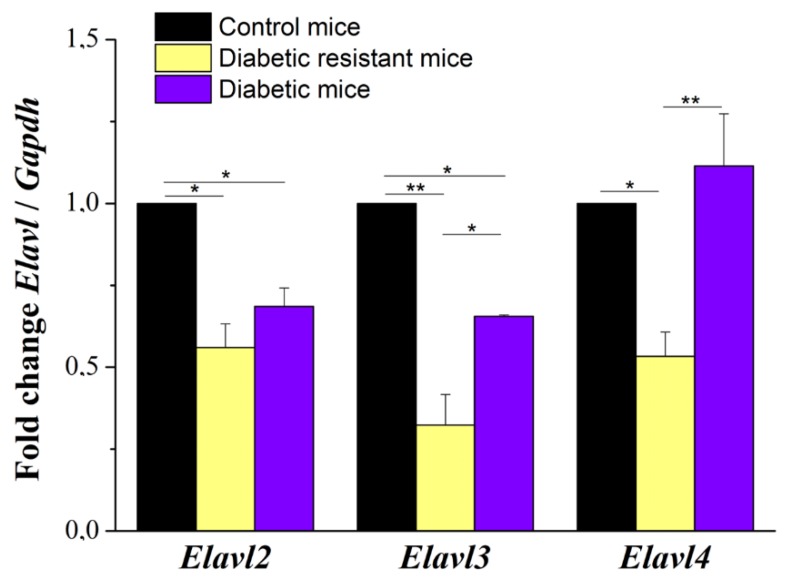
Fold change of *Elav*-like gene expression with respect to *Gapdh* expression in dorsal root ganglia (DRG) neurons of control, diabetic resistant, and diabetic mice. Statistical significance is indicated * *p* < 0.05, ** *p* < 0.01.

The qRT-PCR analysis revealed the decrease of the *Elav*-like gene expression in mouse DRG neurons for all three *Elavl* (i.e., *Elavl2*, *Elavl3,* and *Elavl4*) in diabetic mice and diabetic resistant mice compared to control mice (Figure 4). In the DRG neurons of control mice, we obtained similar levels for *Elavl2, Elavl3,* and *Elavl4* genes (unshown data). 

We demonstrated that *Elavl* genes expression is altered in diabetes, the two-way ANOVA analysis being statistical significant for the *Elavl* expression, for the diabetic condition and for their interaction (Table S9). *Elavl* genes were downregulated in the diabetic condition and strongly downregulated in the diabetic resistant condition in comparison with control. To detail, *Elavl2* expression decreased to 0.68 ± 0.05 fold in diabetic group and to 0.56 ± 0.07 fold in the diabetic resistant group compared to the control group. *Elavl3* expression decreased to 0.65 ± 0.01 fold in the diabetic group and to 0.32 ± 0.09 fold in the diabetic resistant group compared to control group. *Elavl4* expression decreased to 0.53 ± 0.07 fold in the diabetic resistant group compared to the control group, while in the diabetic group there was a tendency of expression increase without significance (Table S10). 

### 2.4. Hu Proteins Expression in Mouse DRG Neurons Is Decreased in Diabetic and Diabetic Resistant Mice Compared to Control Mice

**Figure 5:**
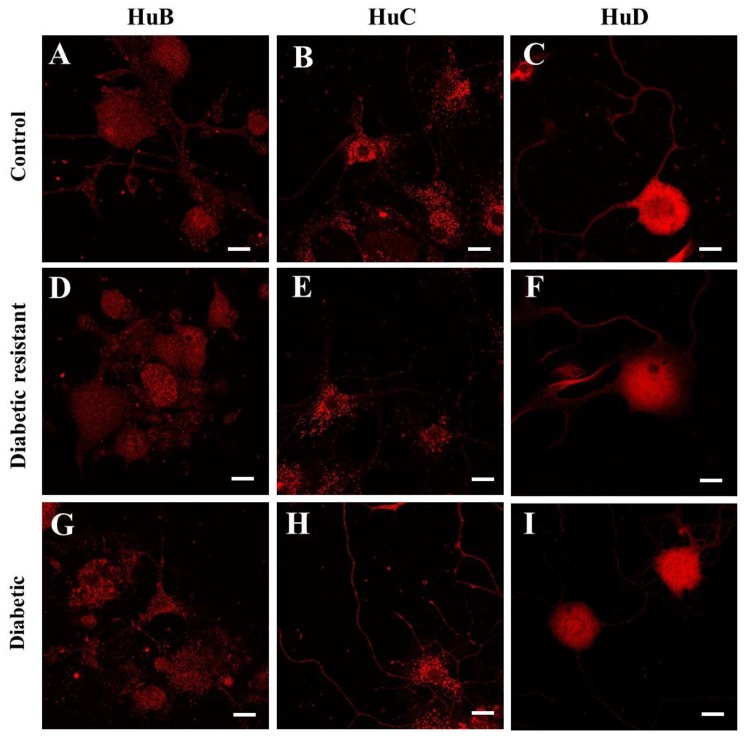
Hu proteins (HuB, HuC, and HuD ) expression in DRG neurons of control (**A**–**C**), diabetic resistant (**D**–**F**), and diabetic mice (**G**–**I**). The red labeling is obtained with rabbit polyclonal anti-ELAVL2, anti-ELAVL3, and anti-ELAVL4 antibodies, respectively, followed by the staining with donkey polyclonal anti-rabbit conjugated with Rhodamine Red X. Images are captured with an LSM 710 Zeiss laser scanning microscope using a 63× oil objective. Scale bar 10 μm.

The Hu protein expression in mouse DRG neurons was evaluated by immunofluorescence in control (Figure 5A–C), diabetic resistant (Figure 5D–F), and diabetic (Figure 5G–I) mice. We evidenced the expression of HuB, HuC, and HuD proteins in DRG neurons for all three CD-1 mice groups. We localized all three Hu proteins both in the soma and the neurites of the DRG neurons. Each Hu protein has a distinct distribution pattern in the soma and particularly HuC tends to organize in clusters. We observe a pronounced localisation of HuB and HuC in the neurites in diabetic conditions compared to diabetic resistant or control conditions.

**Figure 6:**
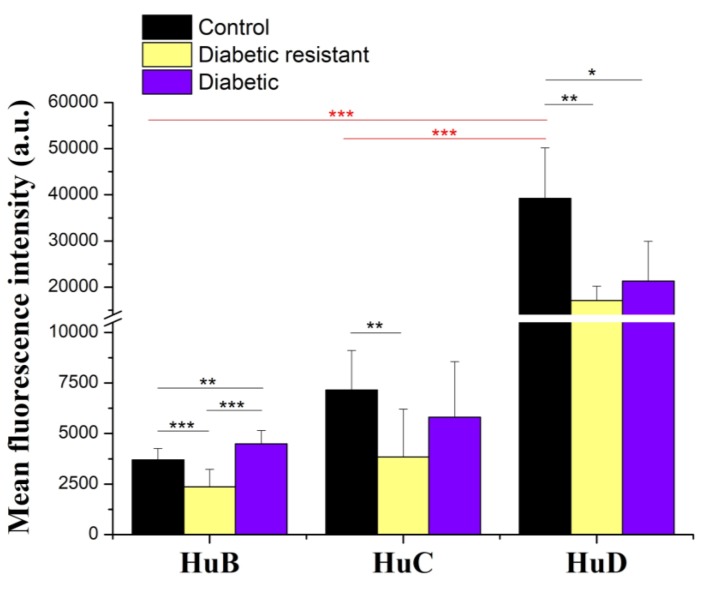
Hu protein expression based on mean fluorescence intensity analysis in DRG neurons of control, diabetic resistant, and diabetic mice. Data are expressed as mean ± SD in the captured images. Statistical significance is indicated * *p* < 0.05, ** *p* < 0.01, *** *p* < 0.001.

Further on, we performed the quantitative analysis of the neuronal Hu proteins expression based on the mean fluorescence intensity (Figure 6) and we correlated these results with the *Elav*-like gene expression. In control mice, we obtained the following ranking for the protein expression HuD > HuC > HuB, and the one-way ANOVA analysis followed by post-hoc Bonferroni test indicated statistical significance between HuD and HuB expression (*p* < 0.001) and between HuD and HuC expression (*p* < 0.001). However, the distinct levels of HuB, HuC, and HuD expression in control mice are not in agreement with the *Elav*-like gene levels that are comparable. In diabetic and diabetic resistant conditions neuronal HuB, HuC, and HuD proteins were distinctly regulated compared to control conditions. The two-way ANOVA analysis indicated statistical significance of Hu proteins expression, for the diabetic condition and for their interaction (Table S11). In comparison with control mice, HuB protein was significanly downregulated in diabetic resistant mice and upregulated in diabetic mice, HuC protein was significantly downregulated in diabetic resistant mice, and HuD protein was significantly downregulated both in diabetic and diabetic resistant mice (Table S12). 

## 3. Discussion

In this study, we brought evidence that Hu proteins undergo expression changes that might be associated with the diabetic condition. First of all, it is necessary to discuss the model of diabetes that we employed in our study. Indeed, several mouse models for type 1 diabetes have been developed, the most employed being the STZ-induced diabetes, despite its variability, depending on the mice strain [34] or the development of diabetic neuropathy [35]. To detail, variable concentrations of STZ (single i.p. injection) were used in different mice strains to induce diabetes, i.e., ICR, ddY and BALB/c: 100–200 mg/kg and C57BL/6: 75–150 mg/kg [34]. Considering the different mice strain sensitivity to STZ-induction of diabetogenic state [35,36,37], in our study, we decided to induce diabetes in CD-1 adult mice with single i.p. STZ injection (150 mg/kg). 

Despite its variability the STZ-induced model of diabetes is robust and used in multiple studies. However, researchers focus either on the mortality rate or on the resistance of the animal strain when injected with STZ, but in the STZ-“sensitive” animal strains little attention is paid to the rate of surviving animals that are resistant to the STZ-induction of diabetes. Some studies reported a subpopulation of mice [37] or rats [38] remains normoglycemic upon STZ-induction of diabetes, but do not explicitly consider these animals as “diabetic resistant”. In our study, we are classifying STZ-resistant CD1-mice as “diabetic resistant” mice. 

The resistance to STZ-induction of diabetes was previously described in different mice strains, including mice lacking phosphatase and a tensin homolog deleted from chromosome 10 [39] or nonobese diabetes-resistant mice [40,41]. On the other hand, the resistance to STZ-induction of diabetes in a certain percentage of animals belonging to the so-called ‘sensitive’ strains (e.g., CD-1 mice) is generally not discussed. For example, in CD-1 mice, the distinction between the induction of type 1 diabetes by single injection of high STZ dose (130 mg/kg or 150 mg/kg) and the induction of type 2 diabetes by multiple injections of low STZ dose (40 mg/kg) was reported [42], but the percentage of 100% reported in the text for the induction of diabetes animals by single injection of high STZ dose is not in agreement with the percentage of animals with hyperglycemia ≥600 mg/dL, one out of five animals (130 mg/kg STZ, 16% mortality) and three out of three (150 mg/kg STZ, 50% mortality) presented in the same study. In our study, we demonstrate the resistance to the induction of diabetes with a single injection of high STZ dose (150 mg/kg) in approximately 46% (CD-1 adult male mice, six out of 13 mice) of the surviving animals (37% mortality). We also evidence that diabetic-resistant mice have lower body weight values compared to control mice, but remain normoglycemic. Our data, indicating that diabetic mice have a lower body weight and a higher glycemia compared to control mice, are in agreement with previous protocols of STZ-induction of diabetes [37]. To resume, our study demonstrates that, in addition to the expected population of STZ-induced hyperglycemic CD-1 mice, a mice subpopulation develops resistance to STZ and we consider that a distinct analysis of the STZ-resistant normoglycemic mice should be done in each study, as the findings might be relevant in understanding the mechanisms of insulin resistance development in human patients. 

In diabetic patients, diabetic peripheral neuropathy is gradually characterized by hyperalgesia and allodynia, followed by the development of hypoalgesia and finally the complete loss of sensation [43]. STZ-induced diabetes in mice or rat is associated with thermal hyperalgesia in early phases [43] and with thermal hypoalgesia in late stages of diabetes [44,45,46] in the absence of insulin therapy. Commonly thermal hypoalgesia precedes epidermal denervation in STZ-diabetic mice [47]. We confirm an increase in the paw withdrawal latency (thermal hypoalgesia) in diabetic mice 8-weeks after STZ-induction of diabetes, while diabetic resistant mice have similar paw withdrawal latencies compared to control mice. The absence of changes in the algesic profile of diabetic resistant mice is supported by previous reports showing that ~50% male Sprague-Dawley rats remain normoglycemic after STZ-injection, without significant changes in the algesic profile (no changes in the threshold or latency to heat noxious stimuli, or in the pressure pain threshold and frequency of withdrawal to brush and 20-g von Frey filament) compared to control rats [38].

Considering previous studies that reported the role played by Hu proteins from DRG neurons in hyperalgesia [19,48,49,50,51,52], we analyzed the expression changes of Hu proteins in diabetic and diabetic resistant mice compared to control mice and correlated these data with algesic profile to radiant heat exposure. To detail, HuR contributes to hyperalgesia either associated with experimental autoimmune encephalomyelitis [48] or with inflammation (exposure to bradykinin and interleukin-1) where stabilized cyclooxygenase-2 mRNA [49]. HuD is upregulated and contributes to pain hypersensitivity to mechanical and cold stimulation in antiretroviral-evoked painful neuropathy by regulating spinal ryanodine receptor-2 [50] or GAP43 [19,51] or contributes to thermal hot hyperalgesia in oxaliplatin-induced neuropathy by regulating GAP43 [52]. On the other hand, to the best of our knowledge, this is the first study documenting the role of Hu proteins in hypoalgesia associated with the diabetic condition, addressing the expression of Hu proteins in DRG neurons. Considering the previous reports regarding the role of *nELAVL* Hu-proteins in neuronal excitability by binding to the mRNAs encoding proteins from the glutamate synthesis pathway [15], or encoding Kv1.1 voltage-gated potassium channels [16], we might suppose that *nELAVL* Hu-proteins might also stabilize / regulate mRNA encoding other proteins (i.e., ion channels) involved in DRG neuronal excitability and being important players in the algesic profile and diabetes. 

Our study brings evidence that *Elavl* genes and Hu proteins expression is distinctly regulated in DRG sensory neurons in diabetic, diabetic resistant, and control conditions, and we have tried to correlate these expression data with the final paw withdrawal latency in the hot plate test. Interestingly, the final paw withdrawal latency in diabetic-resistant mice has not significantly changed in comparison to control mice, which indicates that diabetic-resistance mice do not undergo changes in the algesic profile in radiant heat exposure after 8 weeks. In diabetic mice, *Elavl2* and *Elavl3* are downregulated, while HuB is upregulated and HuD is downregulated, compared to control mice. In diabetic resistant mice, both *Elavl* genes and Hu proteins are strongly downregulated, compared to control mice. It is very interesting to remark that, despite the lack of changes in the algesic profile of diabetic resistant mice, we reported significant *Elavl* gene and Hu protein expression changes in diabetic resistant mice compared to diabetic or control mice. Previous studies indicated HuD upregulation in thermal hyperalgesia [19,50,51,52] and our study brings evidence that HuD is downregulated in thermal hypoalgesia induced by the advanced diabetes status. Considering the role played by HuD upregulation in nerve regeneration upon lesion [21], a possible scenario in diabetes would be: (i) hyperalgesia (early phases of diabetes) is associated with HuD upregulation involved in nerve regeneration, (ii) hypoalgesia (late phases of diabetes) is associated with HuD downregulation, when its ability to regulate mRNA proteins involved in nerve recovery is overcome. However, HuD downregulation in late diabetes should be considered with caution as STZ-induced the same kind of expression changes in normoglycemic diabetic resistant mice. Extensive analysis of the algesic profile in diabetic and diabetic resistant mice in correlation with Hu proteins expression is necessary. 

Our study also analyzed the immunolocalization of Hu proteins in correlation with the diabetic status. Previous immunostaining studies documented the expression of HuD [8,53], HuC/HuD (anti-16A11 antibody) [17], or all Hu proteins (anti-16A11 antibody) [18] in adult DRG neurons. The HuD immunopositivity in DRGs neurons was analyzed: (i) in the cell compartments, with distribution both in the soma and the axons [19] or (ii) in the subcellular structures (strong staining in the cytoplasm [8,18] and low staining in the nucleus, Golgi apparatus and mitochondria [18]). Our study indicates HuB, HuC, and HuD expression in the soma and neurites of the DRG neurons. However, our semi-quantitative analysis of Hu protein expression was limited to the soma of DRG neurons. 

Although specific immunostaining was obtained for all three neuronal Hu proteins in different structures of the central nervous system or in the spinal cord [54], only HuD specific immunopositivity was analyzed in DRGs [8,53], but no specific targeting of HuB and HuC expression in DRGs was done. In our study, we bring evidence of the specific localisation of HuB and HuC in DRG neurons, and we also demonstrate that all three Hu proteins undergo expression changes in late diabetes. 

Different neuronal types from hippocampus, cerebellum, olfactory cortex, neocortex, etc. were demonstrated to express from one to several Hu genes [54]. We might suppose that different subtypes of DRG neurons express various combinations of Hu genes, distinctly contributing to the regulation/stabilization of mRNA encoding proteins involved in the development of diabetic neuropathy and/or thermal hypoalgesia. To this purpose, subsequent colocalization studies of Hu proteins in DRG neurons might bring new insights.

To resume, our study analyzed the distinction between diabetic and diabetic resistant mice in the STZ-induction model and compares them with the control mice. We correlate the diabetic state with hyperglycemia, lower body weight, presence of late thermal hypoalgesia, *Elavl2* and *Elavl3* downregulation, HuB upregulation, and HuD downregulation in comparison to control conditions. Meanwhile, we correlate the diabetic resistant state with normoglycemia, slightly lower body weight, normal algesia, strong *Elavl2, Elavl3,* and *Elavl4* downregulation, HuB, HuC, and HuD downregulation compared to control conditions. In conclusion, we demonstrate the distinct expression regulation of *nELAVL* Hu proteins in diabetes and we consider that it is very important to understand if these Hu protein expression changes are also present in patients with peripheral diabetic neuropathy and if there is any correlation with the status of the disease.

## 4. Materials and Methods

### 4.1. Animals

Adult CD-1 male mice aged 6 weeks with a mean body weight of 20 g were acquired from the “Cantacuzino” Medico-Military National Institute of Research and Development. Animals (*N* = 40) were housed 3/cage in the animal husbandry of ‘Horia Hulubei’ National Institute of Physics and Nuclear Engineering, with food and water *ad libitum*. All procedures were in accordance with the European Guidelines on Laboratory Animal Care, and with the approval of the institutional Ethics Committee of the ’Horia Hulubei’ National Institute of Physics and Nuclear Engineering (approval number 31/11.06.2015).

### 4.2. Streptozotocin-Induced Diabetes

**Figure 7:**
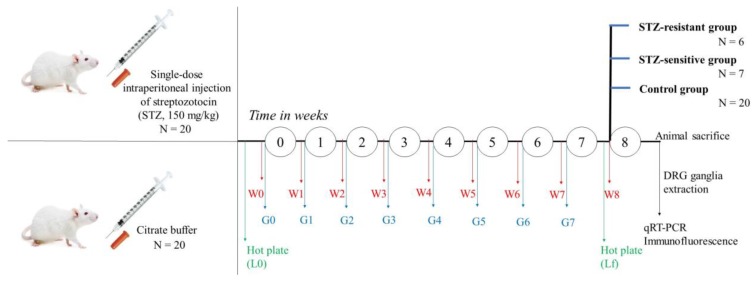
Timeline of the experimental protocol. Abbreviations W-weight, G-glycemia.

Animals were divided into equal groups: 20 mice treated with citrate buffer solution and 20 mice treated with streptozotocin (STZ, #S0130, Sigma-Aldrich, St. Louis, MO, USA). Diabetes was induced with a single intraperitoneal injection of STZ, at a fixed volume of 300 μL/animal, at the final concentration of 150 mg/kg/body weight in 0.05 mol/L sodium citrate buffer, pH 4.5, as previously described [37,55,56]. Citrate buffer solution was also injected intraperitoneal at a fixed volume of 300 μL/animal. Upon data analysis, the surviving animals from the STZ-injected group were divided into two subgroups: STZ-sensitive group and STZ-resistant group (see Results Section 2.1). The timeline of the experimental protocol is presented in Figure 7. 

### 4.3. Body Weight Measurements

The body weight measurement was performed before the intraperitoneal injection (STZ or citrate buffer solution), and then repeated once per week (in the same day, at the beginning of the week, and at the same hour) for 8 weeks, as shown in Figure 2. 

### 4.4. Glycemia Measurements

Blood glucose was measured from the tail vein blood by a glucometer (OneTouch, LifeScan, Milpitas, CA, USA). The blood glucose measurement was performed before the intraperitoneal injection (STZ or citrate buffer solution), and then repeated once per week (in the same day and at the same hour) for 7 weeks, as shown in Figure 1. Animals were fastened 12 h before the glycemia measurement. The body weight measurement was done alternately done before/after the glycemia measurement. In order to prevent any experimental bias, in the 8th week, the glycemia measurement was not performed, as animals were subjected to the hot-plate test. 

### 4.5. Hot-Plate Test

In order to determine the occurrence of thermal hypersensitivity, the mice subgroups (control, STZ-sensitive and STZ-resistant) were subjected to the hot plate test. To detail, CD-1 mice were placed individually on a Hot Plate Analgesia Meter (Ugo Basile, Comerio, Varese, Italy), maintained at 55 °C and the latency to hind paw licking or flicking (whichever occurs first) was measured. The animals that did not respond within 30 s were removed from the hot plate to prevent paw damage. The hot plate test was performed before the intraperitoneal injection with STZ or citrate buffer solution (initial paw withdrawal latency, L0), and repeated after 8 weeks (final paw withdrawal latency, Lf), as shown in Figure 3.

### 4.6. Primary Cultures of Neurons from Dorsal Root Ganglia

Animals were sacrificed after 8 weeks from the intraperitoneal injection (at 16 weeks of age) and DRG neurons were obtained from all spinal levels of adult male CD1 mice as previously described [32,33]. The animals were exposed to CO_2_ inhalation (1 min) followed by decapitation according to the European Guidelines on Laboratory Animal Care, with the approval of the institutional Ethics Committee of the ‘Horia Hulubei’ National Institute of Physics and Nuclear Engineering (approval number, 31/11.06.2015). DRGs were removed under sterile conditions and were immediately transferred into IncMix solution (in mM, NaCl 155, K_2_HPO4 1.5, HEPES 5.6, Na-HEPES 4.8, glucose 5). After cleaning the ganglia from the surrounding tissue and counting them, DRGs were incubated in a mixture of 1 mg/mL Collagenase from *Clostridium histolyticum,* type 1A (#C9891, Sigma-Aldrich, St. Louis, MO, USA) and 1 mg/mL Dispase from *Bacillus polymyxa* (#17105041, GIBCO, Invitrogen, Carlsbad, CA, USA) in IncMix solution for 1 h at 37 °C. Following enzyme treatment, the ganglia were washed once in Dulbecco’s modified Eagle’s medium Ham’s F-12 (DMEM F-12, #D8900, Sigma-Aldrich, St. Louis, MO, USA) with 10% horse serum (#H1270, Sigma-Aldrich, St. Louis, MO, USA), before mechanical trituration in 0.5 mL DMEM F-12. The dissociated neurons were then washed by centrifugation (at 1000× *g* for 10 min, 25 °C) followed by resuspension in fresh medium. Following the final wash, the cell pellet was resuspended in DMEM F-12, containing 10% horse serum and 50 μg/mL gentamicin (#G1272, Sigma-Aldrich, St. Louis, MO, USA). Following a second trituration, neurons were seeded on 13-mm coverslips, previously coated with 1 mg/mL poly-D-lysine (#P0899, Sigma-Aldrich, St. Louis, MO, USA) for 1 h at 37 °C and after 24 h were further processed for the immunostaining protocol. In the case of qRT-PCR protocol, the extracted DRGs were directly subjected to the RNA extraction protocol.

### 4.7. RNA Isolation and Quantitative Real-Time PCR (qRT-PCR)

In order to quantify the expression levels of ELAV2, ELAV3, and ELAV4 in the DRG neurons from STZ-sensitive, STZ-resistant and control CD-1 mice subgroups, the total RNA was extracted from dissociated ganglia using the GenElute Mammalian Total RNA MiniPrep Kit (#RTN70, Sigma-Aldrich, St. Louis, MO, USA) according to the manufacturer’s instructions. RNA concentrations were determined by spectrophotometric measurements at 260 and 280 nm (Beckman Coulter DU 730). In agreement with the manufacturer guidelines (Sigma-Aldrich, St. Louis, MO, USA) for the GenElute™ Mammalian Total RNA Miniprep Kit, in our experiments the A_260_:A_280_ ratio was 2.038 ± 0.07. Reverse transcription was performed using the High-Capacity cDNA Archieve Kit (Applied Biosystems, Foster City, California, USA). The relative abundance of ELAV transcripts was assessed by qRT-PCR using TaqMan methodology and the ABI Prism 7300 Sequence Detection System (Applied Biosystems). Reactions were carried out for 35 cycles in triplicate. *Elavl2* (#Mm00516015_m1), *Elavl3* (#Mm01151962_m1), *Elavl4* (#Mm01263580_mH) and the mouse control assay for glyceraldehydes-3-dehydrogenase (*Gapdh*, #Mm999999_g1) were obtained from Life Technologies (Carlsbad, CA, USA), and used in accordance with manufacturer’s guidelines. From each animal group, 3 animals were sacrificed for the qRT-pCR analysis, and each gene was analyzed in triplicate. Quantitative RT-PCR data for each *Elavl* were normalized with *Gapdh* mRNA levels and relative amounts of mRNA were determined using the comparative cycle thresholds [57].

### 4.8. Immunofluorescence

DRG neurons in primary culture seeded on 13-mm coverslips were washed with PBS, fixed in 4% paraformaldehyde, permeabilized with 0.1% Triton X-100 and immunostained. Non-specific binding was blocked with donkey serum (#017-000-001, Jackson ImmunoResearch Laboratories, UK). DRG neurons were incubated with the primary antibodies overnight at 4 °C. We have used the following primary antibodies: rabbit polyclonal IgG anti-ELAVL2 antibody (1:100; #ab96471, Abcam, Cambridge, UK), rabbit polyclonal IgG anti-ELAVL3 antibody (1:100; #ab78467, Abcam), and rabbit polyclonal IgG anti-ELAVL4 antibody (1:100; #ab96474, Abcam), considering the target specificity of these antibodies described by previous studies [20,25,58,59]. Then, DRG neurons were incubated with the secondary antibody donkey polyclonal anti-rabbit IgG (H+L) conjugated with Rhodamine Red X (1:100; #111-295-003, Jackson ImmunoResearch Laboratories, UK) for 1h at room temperature. The primary antibody was omitted in negative-control samples. Images were captured using a confocal fluorescence microscope (LSM 710, Carl Zeiss, Oberkochen, Germany) equipped with a 63× oil objective. The following acquisition parameters were used: pinhole corresponding to 1 Airy Unit, 62 μm for the 488 nm laser, digital gain of 1.00, and 5% intensity of the laser, as we previously employed [60]. The acquisition parameter settings were kept fixed across all the image acquisition sessions. Image acquisition was carried out using Zeiss LSM Image Browser software (Carl Zeiss, Germany). 

### 4.9. Digital Image Analysis 

The fluorescence images were preprocessed using ImageJ. The outline of each neuronal soma was manually drawn and the quantitative analysis of the fluorescence signal was done (mean pixel intensity), as presviously described [61,62]. This quantification was carried out on both negative control (the primary antibody was omitted in the immunofluorescence protocol) and positive samples (full immunostaining protocol, including primary antibody). The average per group (control, diabetic and diabetic resistant) negative control mean intensity was subtracted from the mean intensity values of positive samples resulting the corrected mean pixel intensity. For each Hu protein, ~30 cells were scored from each mice group. Finally, we plotted the corrected mean pixel intensity calculated for each Hu protein (i.e., HuB, HuC, and HuD ) for the samples obtained from the control, diabetic and diabetic resistant mice group.

### 4.10. Statistical Analysis

Statistical analysis was performed using OriginPro 8 (OriginLab Corporation, Northampton, MA, USA).

Glycemia values were compared by: (i) by two-way ANOVA followed by post-hoc Bonferroni test (for testing the significance of glycemia change, diabetic condition contribution, and of their interaction), (ii) by one-way ANOVA followed by post-hoc Bonferroni test (for testing the inter-group significance of glycemia change for each week after the STZ-induction of diabetes), and (iii) by one-way ANOVA followed by post-hoc Bonferroni test (for testing the intra-group significance of glycemia change between different weeks after the STZ-induction of diabetes).

Body weight values were compared as follows: (i) by two-way ANOVA followed by post-hoc Bonferroni test (for testing the significance of body weight change, diabetic condition contribution and of their interaction), (ii) by one-way ANOVA followed by post-hoc Bonferroni test (for testing the inter-group significance of body weight change for each week after the STZ-induction of diabetes), and (iii) by one-way ANOVA followed by post-hoc Bonferroni test (for testing the intra-group significance of body weight change between different weeks after the STZ-induction of diabetes).

Paw withdrawal latency values obtained in the hot plate test were analyzed as follows: (i) by two-way ANOVA followed by post-hoc Bonferroni test (for testing the significance of final latency change, diabetic condition contribution and of their interaction), (ii) by one-way ANOVA followed by post-hoc Bonferroni test (for testing the inter-group significance of the final latency change), and (iii) by unpaired Student *t*-test (for testing the intra-group differences between the initial and final latency).

Quantitative RT-PCR data were analyzed as follows: (i) by two-way ANOVA followed by post-hoc Bonferroni test (for testing the significance of *Elavl* expression change, diabetic condition contribution and of their interaction) and (ii) by one-way ANOVA followed by post-hoc Bonferroni test (for testing the inter-group significance of *Elavl-2* expression, *Elavl-3* expression or *Elavl-4* expression change).

Mean grey levels obtained by immunofluorescence data analysis were compared as follows: (i) by two-way ANOVA followed by post-hoc Bonferroni test (for testing the significance of Hu protein expression change, diabetic condition contribution and of their interaction), (ii) by one-way ANOVA followed by post-hoc Bonferroni test (for testing the inter-group significance of HuB expression, HuC expression or HuD expression change and (iii) by one-way ANOVA followed by post-hoc Bonferroni test (for testing the intra-group significance of ELAV expression change in control mice). 

Data were represented in OriginPro 8 (OriginLab Corporation, Northampton, MA, USA) as the mean ± SD. Differences were considered significant at *p* < 0.05. Statistical significance is indicated in figures as follows: * *p* < 0.05, ** *p* < 0.01, ** *p* < 0.001.

## Supplementary Materials

Supplementary materials can be found at https://www.mdpi.com/1422-0067/20/8/1965/s1.
