# Supplementary material for: RNA-Binding Proteins HuB, HuC, and HuD are Distinctly Regulated in Dorsal Root Ganglia Neurons from STZ-Sensitive Compared to STZ-Resistant Diabetic Mice
Source: Int J Mol Sci. 2019 Apr 22;20(8):1965. doi: 10.3390/ijms20081965 (PMC6514878; doi:10.3390/ijms20081965)
Supplement: Supplementary file 1 [file ijms-20-01965-s001.pdf]

RNA-Binding Proteins HuB, HuC, and HuD are Distinctly Regulated in Dorsal Root Ganglia Neurons from STZ-Sensitive Compared to STZ-Resistant Diabetic Mice

1. *Statistical analysis of glycemia changes*

Supplementary Table 1. Two-way ANOVA analysis of the glycemia changes between diabetic, diabetic resistant and control groups

Overall tw-way ANOVA			
	**DF**	**F value**	**P**
**Glycemia**	7	60.01	P < 0.001
**Diabetic condition**	2	636.85	P < 0.001
**Interaction**	14	53.54	P < 0.001
Post-hoc Bonferroni test			
	**P**		
**Diabetic resistant vs Control**	P < 0.001		
**Diabetic vs Control**	P < 0.001		
**Diabetic vs Diabetic resistant**	P < 0.001		

Supplementary Table 2. One-way ANOVA analysis – the weekly (Wk) analysis of the glycemia differences between the animal groups

Overall one-way ANOVA			
	**DF**	**F value**	**P**
**Wk0**	2	4.08	P < 0.05
Post-hoc Bonferroni test			
	**P**		
Diabetic resistant vs Control	Ns		
Diabetic vs Control	ns		
Diabetic vs Diabetic resistant	P < 0.05		
Overall one-way ANOVA			
	**DF**	**F value**	**P**
**Wk1**	2	6.12	P < 0.01
Post-hoc Bonferroni test			
	**P**		
Diabetic resistant vs Control	ns		
Diabetic vs Control	P < 0.01		
Diabetic vs Diabetic resistant	ns		
Overall one-way ANOVA			
	**DF**	**F value**	**P**
**Wk2**	2	22.87	P < 0.001
Post-hoc Bonferroni test			
	**P**		

Diabetic resistant vs Control	ns		
Diabetic vs Control	P < 0.001		
Diabetic vs Diabetic resistant	P < 0.001		
Overall one-way ANOVA			
	**DF**	**F value**	**P**
**Wk3**	2	26.10	P < 0.001
Post-hoc Bonferroni test			
	**P**		
Diabetic resistant vs Control	ns		
Diabetic vs Control	P < 0.001		
Diabetic vs Diabetic resistant	P < 0.001		
Overall one-way ANOVA			
	**DF**	**F value**	**P**
**Wk4**	2	12.31	P < 0.001
Post-hoc Bonferroni test			
	**P**		
Diabetic resistant vs Control	ns		
Diabetic vs Control	P < 0.001		
Diabetic vs Diabetic resistant	P < 0.01		
Overall one-way ANOVA			
	**DF**	**F value**	**P**
**Wk5**	2	673.74	P < 0.001
Post-hoc Bonferroni test			
	**P**		
Diabetic resistant vs Control	ns		
Diabetic vs Control	P < 0.001		
Diabetic vs Diabetic resistant	P < 0.001		
Overall one-way ANOVA			
	**DF**	**F value**	**P**
**Wk6**	2	436.72	P < 0.001
Post-hoc Bonferroni test			
	**P**		
Diabetic resistant vs Control	ns		
Diabetic vs Control	P < 0.001		
Diabetic vs Diabetic resistant	P < 0.001		
Overall one-way ANOVA			
	**DF**	**F value**	**P**
**Wk7**	2	780.55	P < 0.001
Post-hoc Bonferroni test			
	**P**		
Diabetic resistant vs Control	P < 0.001		
Diabetic vs Control	P < 0.001		
Diabetic vs Diabetic resistant	P < 0.001		

Supplementary Table 3. One-way ANOVA analysis – the weekly comparison of the glycemia values for the diabetic group

Overall one-way ANOVA

	DF	F value	P
**Control group**	7	5.41	ns
Post-hoc Bonferroni test			
	**P**		
Wk1 vs Wk0	ns		
Wk2 vs Wk0	ns		
Wk2 vs Wk1	ns		
Wk3 vs Wk0	ns		
Wk3 vs Wk1	ns		
Wk3 vs Wk2	ns		
Wk4 vs Wk0	ns		
Wk4 vs Wk1	ns		
Wk4 vs Wk2	ns		
Wk4 vs Wk3	ns		
Wk5 vs Wk0	ns		
Wk5 vs Wk1	ns		
Wk5 vs Wk2	ns		
Wk5 vs Wk3	ns		
Wk5 vs Wk4	ns		
Wk6 vs Wk0	ns		
Wk6 vs Wk1	ns		
Wk6 vs Wk2	ns		
Wk6 vs Wk3	ns		
Wk6 vs Wk4	ns		
Wk6 vs Wk5	ns		
Wk7 vs Wk0	ns		
Wk7 vs Wk1	ns		
Wk7 vs Wk2	ns		
Wk7 vs Wk3	ns		
Wk7 vs Wk4	ns		
Wk7 vs Wk5	ns		
Wk7 vs Wk6	ns		
Overall one-way ANOVA			
	**DF**	**F value**	**P**
**Diabetic resistant group**	7	1.88	ns
Post-hoc Bonferroni test			
	**P**		
Wk1 vs Wk0	ns		
Wk2 vs Wk0	ns		
Wk2 vs Wk1	ns		
Wk3 vs Wk0	ns		
Wk3 vs Wk1	ns		
Wk3 vs Wk2	ns		
Wk4 vs Wk0	ns		
Wk4 vs Wk1	ns		
Wk4 vs Wk2	ns		
Wk4 vs Wk3	ns		
Wk5 vs Wk0	ns		

Wk5 vs Wk1	ns		
Wk5 vs Wk2	ns		
Wk5 vs Wk3	ns		
Wk5 vs Wk4	ns		
Wk6 vs Wk0	ns		
Wk6 vs Wk1	ns		
Wk6 vs Wk2	ns		
Wk6 vs Wk3	ns		
Wk6 vs Wk4	ns		
Wk6 vs Wk5	ns		
Wk7 vs Wk0	ns		
Wk7 vs Wk1	ns		
Wk7 vs Wk2	ns		
Wk7 vs Wk3	ns		
Wk7 vs Wk4	ns		
Wk7 vs Wk5	ns		
Wk7 vs Wk6	ns		
Overall one-way ANOVA			
	**DF**	**F value**	**P**
**Diabetic group**	7	40.22	P < 0.001
Post-hoc Bonferroni test			
	**P**		
Wk1 vs Wk0	ns		
Wk2 vs Wk0	ns		
Wk2 vs Wk1	ns		
Wk3 vs Wk0	ns		
Wk3 vs Wk1	ns		
Wk3 vs Wk2	ns		
Wk4 vs Wk0	ns		
Wk4 vs Wk1	ns		
Wk4 vs Wk2	ns		
Wk4 vs Wk3	ns		
Wk5 vs Wk0	P < 0.001		
Wk5 vs Wk1	P < 0.001		
Wk5 vs Wk2	P < 0.001		
Wk5 vs Wk3	P < 0.001		
Wk5 vs Wk4	P < 0.001		
Wk6 vs Wk0	P < 0.001		
Wk6 vs Wk1	P < 0.001		
Wk6 vs Wk2	P < 0.001		
Wk6 vs Wk3	P < 0.001		
Wk6 vs Wk4	P < 0.001		
Wk6 vs Wk5	ns		
Wk7 vs Wk0	P < 0.001		
Wk7 vs Wk1	P < 0.001		
Wk7 vs Wk2	P < 0.001		
Wk7 vs Wk3	P < 0.001		
Wk7 vs Wk4	P < 0.001		

Wk7 vs Wk5	ns
Wk7 vs Wk6	ns

2. *Statistical analysis of the body weight changes*

Supplementary Table 4. Two-way ANOVA analysis of the body weight changes between diabetic, diabetic resistant and control groups

Overall two-way ANOVA			
	**DF**	**F value**	**P**
**Body weight**	8	28.77	P < 0.001
**Diabetic condition**	2	60.74	P < 0.001
**Interaction**	16	2.70	P < 0.001
Post-hoc Bonferroni test			
	**P**		
**Diabetic resistant vs Control**	P < 0.001		
**Diabetic vs Control**	P < 0.001		
**Diabetic vs Diabetic resistant**	ns		

Supplementary Table 5. One-way ANOVA analysis – the weekly (Wk) analysis of the body weight differences between the animal groups

Overall one-way ANOVA			
	**DF**	**F value**	**P**
**Wk0**	2	0.64	ns
Post-hoc Bonferroni test			
	**P**		
Diabetic resistant vs Control	ns		
Diabetic vs Control	ns		
Diabetic vs Diabetic resistant	ns		
Overall one-way ANOVA			
	**DF**	**F value**	**P**
**Wk1**	2	4.77	ns
Post-hoc Bonferroni test			
	**P**		
Diabetic resistant vs Control	ns		
Diabetic vs Control	ns		
Diabetic vs Diabetic resistant	ns		
Overall one-way ANOVA			
	**DF**	**F value**	**P**
**Wk2**	2	3.34	ns
Post-hoc Bonferroni test			
	**P**		
Diabetic resistant vs Control	ns		
Diabetic vs Control	ns		
Diabetic vs Diabetic resistant	ns		

Overall one-way ANOVA			
	**DF**	**F value**	**P**
**Wk3**	2	5.22	P < 0.05
Post-hoc Bonferroni test			
	**P**		
Diabetic resistant vs Control	P < 0.05		
Diabetic vs Control	ns		
Diabetic vs Diabetic resistant	ns		
Overall one-way ANOVA			
	**DF**	**F value**	**P**
**Wk4**	2	10.96	P < 0.001
Post-hoc Bonferroni test			
	**P**		
Diabetic resistant vs Control	P < 0.001		
Diabetic vs Control	P < 0.01		
Diabetic vs Diabetic resistant	ns		
Overall one-way ANOVA			
	**DF**	**F value**	**P**
**Wk5**	2	19.79	P < 0.001
Post-hoc Bonferroni test			
	**P**		
Diabetic resistant vs Control	P < 0.001		
Diabetic vs Control	P < 0.001		
Diabetic vs Diabetic resistant	Ns		
Overall one-way ANOVA			
	**DF**	**F value**	**P**
**Wk6**	2	10.86	P < 0.001
Post-hoc Bonferroni test			
	**P**		
Diabetic resistant vs Control	P < 0.001		
Diabetic vs Control	P < 0.05		
Diabetic vs Diabetic resistant	ns		
Overall one-way ANOVA			
	**DF**	**F value**	**P**
**Wk7**	2	13.71	P < 0.001
Post-hoc Bonferroni test			
	**P**		
Diabetic resistant vs Control	P < 0.001		
Diabetic vs Control	P < 0.01		
Diabetic vs Diabetic resistant	Ns		
Overall one-way ANOVA			
	**DF**	**F value**	**P**
**Wk8**	2	9.94	P < 0.001
Post-hoc Bonferroni test			
	**P**		
Diabetic resistant vs Control	P < 0.01		
Diabetic vs Control	P < 0.05		
Diabetic vs Diabetic resistant	ns		

Supplementary Table 6. One-way ANOVA analysis – the weekly comparison of the body weight values for the control group, diabetic resistant group and diabetic group

Overall one-way ANOVA			
	**DF**	**F value**	**P**
**Control group**	8	56.96	P < 0.001
Post-hoc Bonferroni test			
	**P**		
Wk1 vs Wk0	P < 0.001		
Wk2 vs Wk0	P < 0.001		
Wk2 vs Wk1	ns		
Wk3 vs Wk0	P < 0.001		
Wk3 vs Wk1	ns		
Wk3 vs Wk2	ns		
Wk4 vs Wk0	P < 0.001		
Wk4 vs Wk1	P < 0.001		
Wk4 vs Wk2	P < 0.001		
Wk4 vs Wk3	ns		
Wk5 vs Wk0	P < 0.001		
Wk5 vs Wk1	P < 0.001		
Wk5 vs Wk2	P < 0.001		
Wk5 vs Wk3	P < 0.001		
Wk5 vs Wk4	ns		
Wk6 vs Wk0	P < 0.001		
Wk6 vs Wk1	P < 0.001		
Wk6 vs Wk2	P < 0.001		
Wk6 vs Wk3	P < 0.001		
Wk6 vs Wk4	P < 0.001		
Wk6 vs Wk5	ns		
Wk7 vs Wk0	P < 0.001		
Wk7 vs Wk1	P < 0.001		
Wk7 vs Wk2	P < 0.001		
Wk7 vs Wk3	P < 0.001		
Wk7 vs Wk4	P < 0.001		
Wk7 vs Wk5	ns		
Wk7 vs Wk6	ns		
Wk8 vs Wk0	P < 0.001		
Wk8 vs Wk1	P < 0.001		
Wk8 vs Wk2	P < 0.001		
Wk8 vs Wk3	P < 0.001		
Wk8 vs Wk4	P < 0.001		
Wk8 vs Wk5	ns		
Wk8 vs Wk6	ns		
Wk8 vs Wk7	ns		
Overall one-way ANOVA			
	**DF**	**F value**	**P**

**Diabetic resistant group**	8	5.26	P < 0.001
Post-hoc Bonferroni test			
	**P**		
Wk1 vs Wk0	ns		
Wk2 vs Wk0	ns		
Wk2 vs Wk1	ns		
Wk3 vs Wk0	ns		
Wk3 vs Wk1	ns		
Wk3 vs Wk2	ns		
Wk4 vs Wk0	ns		
Wk4 vs Wk1	ns		
Wk4 vs Wk2	ns		
Wk4 vs Wk3	ns		
Wk5 vs Wk0	ns		
Wk5 vs Wk1	ns		
Wk5 vs Wk2	ns		
Wk5 vs Wk3	ns		
Wk5 vs Wk4	ns		
Wk6 vs Wk0	P < 0.05		
Wk6 vs Wk1	ns		
Wk6 vs Wk2	ns		
Wk6 vs Wk3	ns		
Wk6 vs Wk4	ns		
Wk6 vs Wk5	ns		
Wk7 vs Wk0	P < 0.01		
Wk7 vs Wk1	ns		
Wk7 vs Wk2	ns		
Wk7 vs Wk3	ns		
Wk7 vs Wk4	ns		
Wk7 vs Wk5	ns		
Wk7 vs Wk6	ns		
Wk8 vs Wk0	P < 0.001		
Wk8 vs Wk1	P < 0.01		
Wk8 vs Wk2	P < 0.05		
Wk8 vs Wk3	ns		
Wk8 vs Wk4	ns		
Wk8 vs Wk5	ns		
Wk8 vs Wk6	ns		
Wk8 vs Wk7	ns		
Overall one-way ANOVA			
	**DF**	**F value**	**P**
**Diabetic group**	8	3.44	P < 0.01
Post-hoc Bonferroni test			
	**P**		
Wk1 vs Wk0	ns		
Wk2 vs Wk0	ns		
Wk2 vs Wk1	ns		
Wk3 vs Wk0	ns		

Wk3 vs Wk1	ns
Wk3 vs Wk2	ns
Wk4 vs Wk0	ns
Wk4 vs Wk1	ns
Wk4 vs Wk2	ns
Wk4 vs Wk3	ns
Wk5 vs Wk0	ns
Wk5 vs Wk1	ns
Wk5 vs Wk2	ns
Wk5 vs Wk3	ns
Wk5 vs Wk4	ns
Wk6 vs Wk0	ns
Wk6 vs Wk1	ns
Wk6 vs Wk2	ns
Wk6 vs Wk3	ns
Wk6 vs Wk4	ns
Wk6 vs Wk5	ns
Wk7 vs Wk0	P < 0.05
Wk7 vs Wk1	ns
Wk7 vs Wk2	ns
Wk7 vs Wk3	ns
Wk7 vs Wk4	ns
Wk7 vs Wk5	ns
Wk7 vs Wk6	ns
Wk8 vs Wk0	P < 0.01
Wk8 vs Wk1	ns
Wk8 vs Wk2	ns
Wk8 vs Wk3	ns
Wk8 vs Wk4	ns
Wk8 vs Wk5	ns
Wk8 vs Wk6	ns
Wk8 vs Wk7	ns

3. *Statistical analysis for nociceptive hot thermal stimulation changes*

Supplementary Table 7. Two-way ANOVA analysis of the paw withdrawal latency changes between diabetic, diabetic resistant and control groups

Overall two-way ANOVA			
	**DF**	**F value**	**P**
**Latency**	1	25.18	P < 0.001
**Diabetic condition**	2	28.70	P < 0.001
**Interaction**	2	22.94	P < 0.001
Post-hoc Bonferroni test			
	**P**		
**Diabetic resistant vs Control**	ns		
**Diabetic vs Control**	P < 0.001		
**Diabetic vs Diabetic resistant**	P < 0.001		

Supplementary Table 8. One-way ANOVA analysis of the final paw withdrawal latency (Lf) changes between diabetic, diabetic resistant and control groups

Overall one-way ANOVA			
	**DF**	**F value**	**P**
**Final latency (Lf)**	2	39.38	P < 0.001
Post-hoc Bonferroni test			
	**P**		
Diabetic resistant vs Control	ns		
Diabetic vs Control	P < 0.001		
Diabetic vs Diabetic resistant	P < 0.001		

4. *Statistical analysis for Elav-like gene expression changes determined by qRT-PCR*

Supplementary Table 9. Two-way ANOVA analysis of the *Elav*-like gene expression changes between diabetic, diabetic resistant and control groups

Overall two-way ANOVA			
	**DF**	**F value**	**P**
***Elav*-like gene expression**	2	15.01	P < 0.001
**Diabetic condition**	2	87.93	P < 0.001
**Interaction**	4	8.09	P < 0.01
Post-hoc Bonferroni test			
	**P**		
**Diabetic resistant vs Control**	P < 0.001		
**Diabetic vs Control**	P < 0.01		
**Diabetic vs Diabetic resistant**	P < 0.001		
	**P**		
***Elavl*3 vs *Elavl*2**	P < 0.05		
***Elavl*4 vs *Elavl*2**	ns		
***Elavl*4 vs *Elavl*3**	P < 0.001		

Supplementary Table 10. One-way ANOVA analysis of the expression changes of each *Elav*-like gene between diabetic, diabetic resistant and control groups

Overall one-way ANOVA			
	**DF**	**F value**	**P**
Elavl2	2	36.78	P < 0.01
Post-hoc Bonferroni test			
	**P**		
Diabetic resistant vs Control	P < 0.05		
Diabetic vs Control	P < 0.05		
Diabetic vs Diabetic resistant	ns		
Overall one-way ANOVA			
	**DF**	**F value**	**P**
Elavl3	2	63.77	P < 0.001
Post-hoc Bonferroni test			
	**P**		
Diabetic resistant vs Control	P < 0.001		

Diabetic vs Control	P < 0.05		
Diabetic vs Diabetic resistant	P < 0.05		
Overall one-way ANOVA			
	**DF**	**F value**	**P**
Elavl4	2	26.47	P < 0.01
Post-hoc Bonferroni test			
	**P**		
Diabetic resistant vs Control	P < 0.05		
Diabetic vs Control	ns		
Diabetic vs Diabetic resistant	P < 0.01		

5. *Statistical analysis of Hu proteins expression changes determined by semi-quantitative analysis of immunofluorescence data*

Supplementary Table 11. Two-way ANOVA analysis of the Hu proteins expression changes between diabetic, diabetic resistant and control groups

Overall two-way ANOVA			
	**DF**	**F value**	**P**
**Hu protein expression**	2	188.10	P < 0.001
**Diabetic condition**	2	40.45	P < 0.001
**Interaction**	4	20.79	P < 0.001
Post-hoc Bonferroni test			
	**P**		
**Diabetic resistant vs Control**	P < 0.001		
**Diabetic vs Control**	P < 0.001		
**Diabetic vs Diabetic resistant**	ns		
	**P**		
**HuC vs HuB**	P < 0.001		
**HuD vs HuB**	P < 0.001		
**HuD vs HuC**	P < 0.001		

Supplementary Table 12. One-way ANOVA analysis of the expression changes for each Hu protein between diabetic, diabetic resistant and control groups

Overall one-way ANOVA			
	**DF**	**F value**	**P**
**HuB**	2	33.26	P < 0.01
Post-hoc Bonferroni test			
	**P**		
Diabetic resistant vs Control	P < 0.001		
Diabetic vs Control	P < 0.01		
Diabetic vs Diabetic resistant	P < 0.001		
Overall one-way ANOVA			
	**DF**	**F value**	**P**
**HuC**	2	7.12	P < 0.01
Post-hoc Bonferroni test			
	**P**		

Diabetic resistant vs Control	P < 0.01		
Diabetic vs Control	ns		
Diabetic vs Diabetic resistant	ns		
Overall one-way ANOVA			
	**DF**	**F value**	**P**
**HuD**	2	10.15	P < 0.01
Post-hoc Bonferroni test			
	**P**		
Diabetic resistant vs Control	P < 0.01		
Diabetic vs Control	P < 0.05		
Diabetic vs Diabetic resistant	ns		
